# Determinants and effects of microvascular obstruction on serial change in left ventricular diastolic function after reperfused acute myocardial infarction

**DOI:** 10.3389/fcvm.2024.1338940

**Published:** 2024-04-26

**Authors:** Hyemoon Chung, Jiwon Seo, In-Soo Kim, Chul Hwan Park, Jong-Youn Kim, Pil-Ki Min, Young Won Yoon, Byoung Kwon Lee, Tae-Hoon Kim, Bum-Kee Hong, Se-Joong Rim, Hyuck Moon Kwon, Eui-Young Choi

**Affiliations:** ^1^ Division of Cardiology, Department of Internal Medicine, Kyung Hee University School of Medicine, Seoul, Republic of Korea; ^2^ Division of Cardiology, Gangnam Severance Hospital, Yonsei University College of Medicine, Seoul, Republic of Korea; ^3^ Department of Radiology, Gangnam Severance Hospital, Yonsei University College of Medicine, Seoul, Republic of Korea

**Keywords:** acute myocardial infarction, systolic function, diastolic function, microvascular obstruction, cardiovascular magnetic resonance

## Abstract

**Background:**

Although left ventricular (LV) diastolic dysfunction is more related to functional capacity after acute myocardial infarction (AMI), the determinants of LV diastolic functional change after reperfused AMI remain unknown. This study aimed to investigate the effects of microvascular obstruction (MVO) on mid-term changes in LV diastolic function after reperfused AMI.

**Methods:**

In a cohort of 72 AMI patients who underwent successful revascularization, echocardiography and cardiovascular magnetic resonance imaging were repeated at 9-month intervals. The late gadolinium enhancement (LGE) amount, segmental extracellular volume fraction, global LV, and left atrial (LA) phasic functions, along with mitral inflow and tissue Doppler measurements, were repeated.

**Results:**

Among the included patients, 31 (43%) patients had MVO. During the 9-month interval, LV ejection fraction (EF) and LV global longitudinal strain (GLS) were significantly improved in accordance with a decrease in LGE amount (from 18.2 to 10.3 g, *p* < 0.001) and LV mass. The deceleration time (DT) of early mitral inflow (188.6 ms–226.3 ms, *p* < 0.001) and LV elastance index (Ed; 0.133 1/ml–0.127 1/ml, *p* = 0.049) were significantly improved, but not in conventional diastolic functional indexes. Their improvements occurred in both groups; however, the degree was less prominent in patients with MVO. The degree of decrease in LGE amount and increase in LVEF was significantly correlated with improvement in LV-Ed or LA phasic function, but not with conventional diastolic functional indexes.

**Conclusions:**

In patients with reperfused AMI, DT of early mitral inflow, phasic LA function, and LV-Ed were more sensitive diastolic functional indexes. The degree of their improvement was less prominent in patients with MVO.

## Introduction

Ischemic heart disease is one of the most common causes of heart failure with reduced left ventricular (LV) ejection fraction (EF). Therefore, one of the main goals of early revascularization in acute myocardial infarction (AMI) patients is to save viable myocardium by preventing infarction expansion and progressive remodeling (1), with the ultimate goal of improving both LV systolic and diastolic function. Compared to chronic progressive diseases, such as various cardiomyopathies, valvular disease, or hypertensive heart disease, AMI is characterized by abrupt alterations in regional LV myocardial function and LV filling. There have been a few debates regarding changes in infarct size in late gadolinium enhancement (LGE) imaging (2) after successful revascularization in patients with AMI since current LGE imaging techniques have some limitations in accurately differentiating scar and adjacent salvageable myocardium. However, several previous studies have shown that LGE amounts tend to decrease over time (3). The presence of microvascular obstruction (MVO) is a well-known barrier to reverse remodeling or a decrease in the amount of LGE (3, 4). However, previous studies have failed to reveal a relationship between changes in infarct size and changes in LV systolic and diastolic function. Moreover, it remains unclear whether a decrease in infarct size translates into wall motion recovery and improvement in chamber diastolic function. Therefore, in the present study, we sought to evaluate the effects of MVO on serial changes in infarct size and extracellular volume fraction (ECV) after reperfused AMI using cardiovascular magnetic resonance imaging (CMR) over 9 months. In addition, repeated comprehensive measurements of LV systolic and diastolic functional indexes, along with LA phasic function using feature tracking CMR and echo-Doppler techniques, were performed to identify mutual relationships with changes in infarct size.

## Materials and methods

### Patient selection

**Figure 1:**
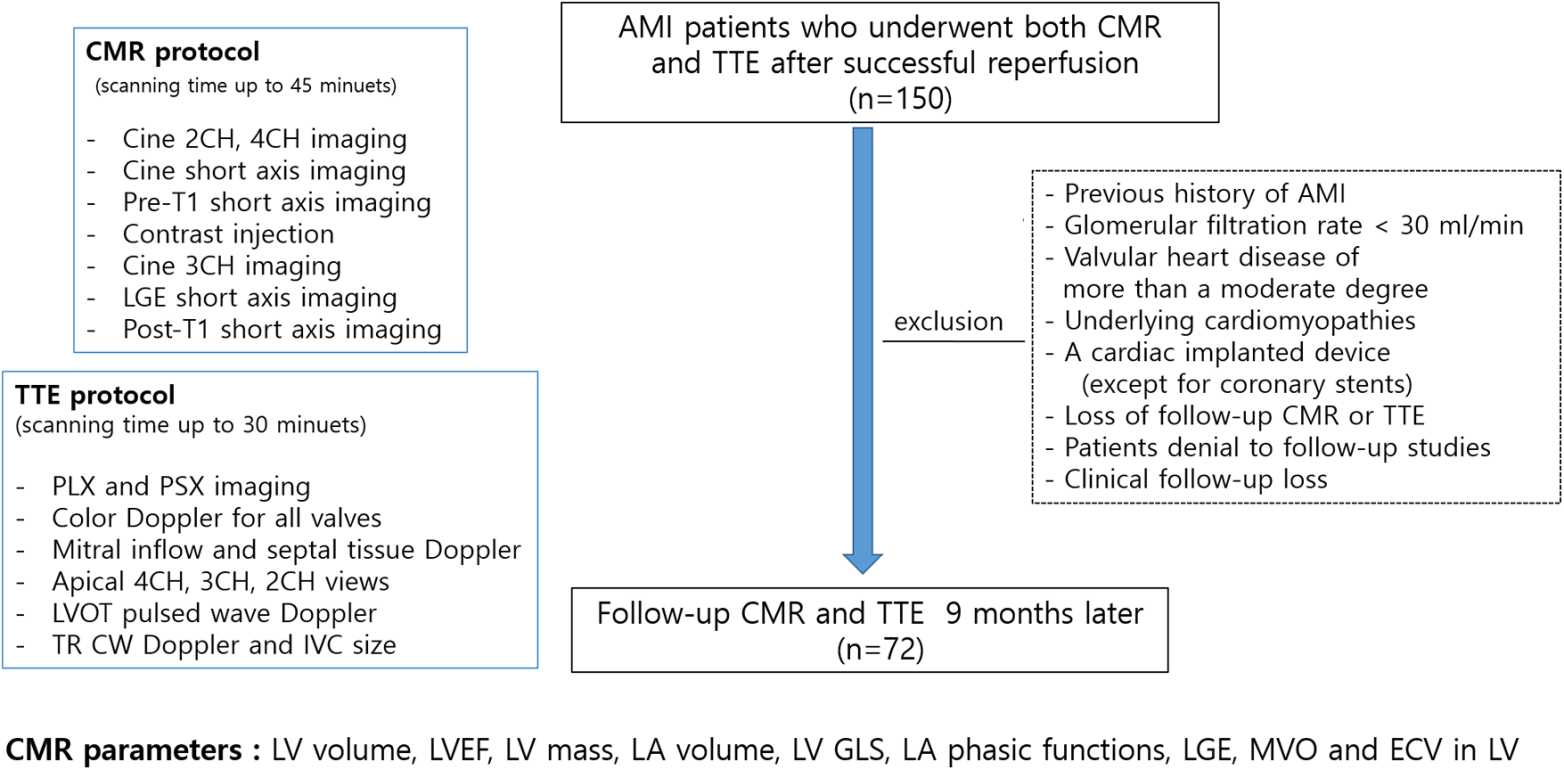
Study flow, imaging protocols and measurements; AMI, acute myocardial infarction; CMR, cardiovascular magnetic resonance; TTE, transthoracic echocardiography; CH, chamber; LGE, late gadolinium enhancement; PLX, parasternal long axis; PSX, parasternal short axis; LV, left ventricular; LVOT, LV outflow tract; TR, tricuspid regurgitant; CW, continuous wave; IVC, inferior vena cava.

Patients with AMI who underwent successful percutaneous coronary intervention (PCI) were prospectively enrolled. The study participants were enrolled from 2012 to 2015 and followed up 9 months later in a tertiary university hospital in Seoul, Republic of Korea. AMI was diagnosed based on elevated levels of cardiac enzymes and ST-segment or T-wave deviation on electrocardiography (ECG) according to the established diagnostic criteria (5). The exclusion criteria were as follows: patients with a previous history of myocardial infarction, claustrophobia, estimated glomerular filtration rate <30 ml/min, valvular heart disease of more than a moderate degree, underlying cardiomyopathies, a cardiac implanted device (except for coronary stents), and poor-quality LGE. Initial CMR was performed on an average of 2.4 ± 2.5 days after revascularization, and all patients underwent echocardiography within 7 days of CMR. Daily ECG follow-up was conducted, and cardiac biomarkers were assessed after admission. The 72 included patients underwent both CMR and echocardiography during the 9-month follow-up. Study flow and protocols are illustrated in Figure 1. The study protocol was approved by the Institutional Review Board of Gangnam Severance Hospital (3-2011-0203), and informed consent was obtained from all participants. The study was performed in accordance with relevant guidelines/regulations and the Declaration of Helsinki.

### CMR

CMR was performed using a 1.5T scanner (Magnetom Avanto, Siemens Medical Solutions, Erlangen, Germany) with a phased-array body coil. The LV two-, three-, and four-chamber and short-axis views were obtained using cine images with a steady-state free precession sequence. The acquisition parameters were as follows: repetition time (TR), 55 ms; echo time (TE), 1.1 ms; flip angle, 67°; 25 phases; slice thickness, 8 mm; slice gap, 2 mm; acquisition matrix, 192 × 109; and field of view, 320 × 400 mm. LGE imaging, with a magnitude- and phase-sensitive inversion recovery prepared fast gradient echo sequence, was obtained 10 min after the administration of 0.2 mmol/kg of a gadolinium-based contrast agent (gadoterate dimeglumine; Dotarem, Guerbet, France). LGE imaging was performed for the same axis and thickness used in the cine imaging. A bolus of contrast medium was intravenously administered at 2 ml/s, followed by 20 ml of normal saline at 4 ml/s through a 20-gauge cannula in the antecubital vein using a power injector (Nemoto; Nemoto Kyorindo, Tokyo, Japan). The appropriate inversion time before LGE imaging was determined using a fast gradient echo sequence with inversion times varying from 150 ms to 650 ms to null the signal from the normal myocardium. The LGE imaging parameters were as follows: TR = 600 ms, TE = 3.4 ms, flip angle = 25°, acquisition matrix = 256 × 156, and field of view = 320 × 400 mm (6). Native T1 mapping with a modified Look-Locker technique was performed during the mid-diastolic phase, and post-T1 mapping was performed 15 min after contrast media injection using the same slice axis and parameters as for the pre-T1 mapping. The scanning planes of T1 mapping were the same as the cine and LGE short-axis image planes. A motion correction algorithm provided by the vendor was used to reduce motion artifacts. The left ventricle was divided into 16 regional segments according to the Society for Cardiovascular Magnetic Resonance guidelines, and the average thickness within each segment was measured (7).

### Measurement of LV mass and extent of LGE

**Figure 2:**
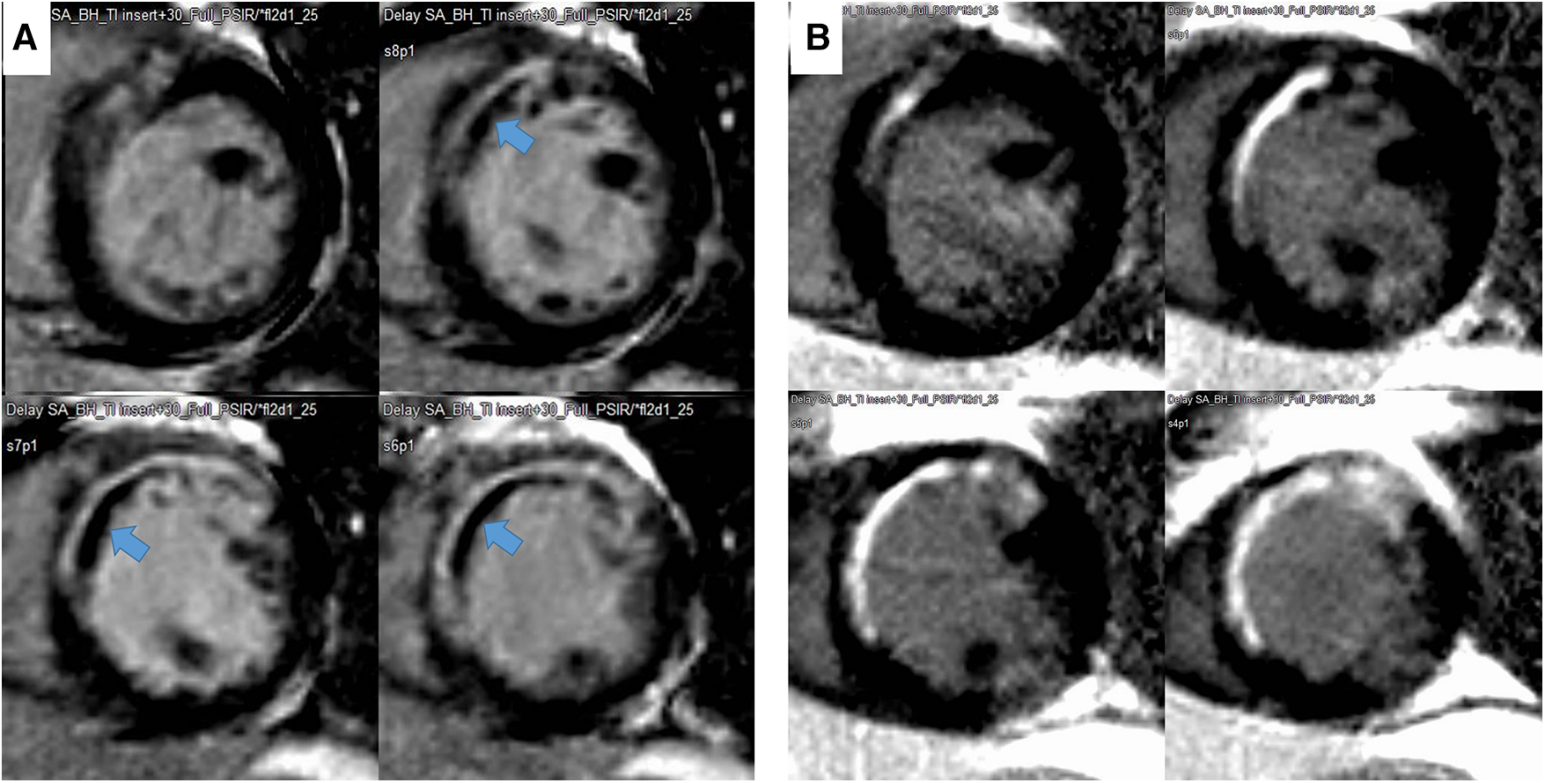
Representative case of a decrease in infarct size and disappearance of microvascular obstruction (MVO) during follow-up. The MVO (black within white, indicated with an arrow) within the late gadolinium enhancement area in the initial image (**A**) and change to LGE during the 9-month follow-up (**B**).

The endocardial and epicardial borders were contoured using a semiautomated method (QMassMR version 8.1, Leiden, Netherlands). To determine the end-diastolic LV mass, the difference between the epicardial and endocardial areas for all slices was multiplied by the slice thickness and section gap and then multiplied by the specific gravity of the myocardium (1.05 g/ml). Papillary muscle mass was included in the LV cavity and excluded from the LV mass measurements. The endocardial areas for all short-axis slices were measured at the end diastole and end systole. From the LGE images, the LV was divided into 16 segments. MVO was defined as a hypoenhanced region in the infarct-related myocardium (Figure 2). The MVO lesion was included in the LGE area. The extent of myocardial scarring, defined as the absolute amount of LGE (g) and percentage of LGE, was measured using dedicated quantitative analysis software (QMassMR version 8.1, Medis, Leiden, Netherlands). The MVO area and its percentage were also automatically measured within the LGE area. In each short-axis slice image, the boundaries of contrast-enhanced areas were automatically traced (using a full-width at half-maximum method that defines the enhanced area by using 50% of the maximum signal found within the enhanced area) (8). The maximum signal was determined using computer-assisted window thresholding of the enhanced area. Obvious artifacts, such as those caused by motion, were excluded by highlighting them using a tool from the software package. Other small isolated regions of enhancement that were not of ischemic origin were also excluded from the analysis. The total infarct size was calculated by the summation of all slice volumes of enhancement, and a 16-segment model was used.

### Chamber performance measured using feature tracking analysis of the left ventricle and left atrium

A myocardial strain analysis using feature tracking CMR was performed using a semiautomated software (Qstrain MR 2.0, Medis, Leiden, Netherlands). The LV endocardial borders were manually drawn in a reference frame. The LV endocardial and epicardial borders were manually traced in two-, three-, and four-chamber long-axis views at the end-systolic and end-diastolic phases, and the LV end-diastolic volume (LVEDV) and LV end-systolic volume (LVESV) were measured. Stroke volume was calculated as LVEDV minus LVESV, and LVEF was calculated as (100 × stroke volume) / LVEDV. The LV mass index was calculated using LV mass / body surface area. LV global longitudinal strain (GLS) was obtained by averaging the longitudinal strains of the apical two-, three-, and four-chamber views. The LA endocardial border was manually traced in a four-chamber long-axis view using LV end diastole as the reference phase. LA maximal, pre-contraction (pre-A in cases without AF), and minimal volumes were obtained from the volume curves generated using Simpson's method (9). LA total emptying fraction, reservoir fraction, conduit fraction, and LA active emptying fraction were also calculated (10). LA-GLS was defined as the average peak strain value. All of the calculation formulas for each parameter are described in Supplementary Table S1.

### Measurement of native T1 and ECV

With QMap and QECV-RE (Medis, Leiden, Netherland), native T1, post-T1, and ECV analyses were repeated in 46 patients. Myocardial ECV was automatically calculated with the following equation (11):

$$\begin{array}{rl} & \mathrm{ECV}=(\Delta R1\;\mathrm{of}\;\mathrm{myocardium}/\Delta R1\;\mathrm{of}\;\mathrm{LV}\;\mathrm{blood}\;\mathrm{pool})\times (1\text{-}\mathrm{hematocrit}),\\ & \mathrm{where}\;\;R1=1/T1\;\mathrm{and}\;\Delta R1=\mathrm{post}\text{-}\mathrm{contrast}\;R1\text{-}\mathrm{pre}\text{-}\mathrm{contrast}\;R1. \end{array}$$

### Conventional echocardiography (supplemental method)

For conventional diastolic functional grade, e′, E/e′, tricuspid regurgitant velocity (TRV), and LA volume index were used. Additionally, the E/A ratio and deceleration time (DT) of early mitral inflow were measured. LV end-diastolic elastance (LV-Ed) was calculated as described in Supplementary Table S1 (12, 13).

### Statistical analysis

Clinical characteristics, echocardiographic parameters, and CMR parameters are presented as mean ± standard deviation with normal distribution or median (interquartile range) without normal distribution for continuous variables and number (percentage) for categorical variables. A correlation analysis between continuous variables was performed using Pearson's correlation coefficient. A paired *t*-test was performed to determine significant changes in variables during the 9-month follow-up. Comparisons of three tertiles, divided by the change in LVEF and LV-GLS, were performed by ANOVA with *post hoc* analysis using the LSD method. All analyses were performed using SPSS (version 25.0, IBM, USA), and statistical significance was set at *p* < 0.05.

## Results

### Baseline characteristics

The average age of the patients was 55 ± 12 years, and 68 (94%) patients were male. The culprit vessel of 49 (68%) patients was the left anterior descending artery (LAD). The median time to reperfusion was 120 (82.5–262.5) min. Fifty-nine (82%) patients took any angiotensin II receptor blockers or angiotensin-converting enzyme inhibitors, and 61 (85%) patients took any beta-blockers. All of them took any anti-platelet agents and statin at discharge. The baseline clinical characteristics are described in Supplementary Table S2. Three patients (4.2%) had grade II diastolic dysfunction, and the rest had grade I diastolic dysfunction according to the guidelines (14, 15). MVO was detected in 31 (43%) patients, and their average percentage of LV mass and absolute value was 4.0 ± 5.5% and 4.8 ± 7.2 g, respectively. Patients with MVO had significantly higher peak CK-MB [200.6 (69.5–364.2) vs. 84.9 (28.7–163.5) ug/L, *p* < 0.001] and % LGE [26.4 (20.9–34.0) vs. 12.9 (4.6–22.3) %, *p* < 0.001] and lower LVEF (44.4 ± 10.9 vs. 49.7 ± 8.0%, *p* = 0.018). Time to reperfusion tended to be shorter and younger in patients with MVO, albeit non-significant. MVO was present in 22 (45%) patients with LAD territory infarction and in 9 (39%) patients with non-LAD territory infarctions. Patients with MVO had lower LA total emptying, reservoir, active emptying fractions, and shorter DT, along with higher LGE amounts. The average ECV of LGE-involved segments was significantly higher in patients with MVO. However, there was no significant difference in medications between the two groups. The baseline characteristics and their comparisons between the MVO groups are described in Tables 1 and 2.

**Table 1:** Baseline characteristics and changes in left ventricular structural and functional indexes during the 9-month follow-up.

	Baseline	Follow-up	*p*
LV ejection fraction, %	47.4 ± 9.6	51.5 ± 9.5	0.001
LVEDV, ml	159.0 ± 30.3	153.1 ± 30.1	0.023
LVESV, ml	84.4 ± 25.4	76.7 ± 23.6	<0.001
LV mass, g	124.2 (107.0–147.2)	105.9 (84.3–119.7)	<0.001
LV-GLS, %	−13.6 ± 4.2	−15.0 ± 4.3	<0.001
Regional LS at initial LGE, %	−16.6 ± 4.9	−18.3 ± 5.1	0.001
Regional LS at initial non-LGE, %	−21.0 ± 6.0	−21.3 ± 6.1	0.624
Echo-Doppler indexes			
E, cm/s	66.2 ± 20.1	65.5 ± 17.9	0.788
A, cm/s	66.0 (54.5–79.5)	64.7 (54.5–77.0)	0.612
DT, ms	188.6 ± 47.3	226.3 ± 54.3	<0.001
E/A ratio	1.02 ± 0.36	1.04 ± 0.41	0.711
s′, cm/s	7.0 (6.0–8.0)	7.3 (7.0–9.0)	0.034
e′, cm/s	6.0 (5.0–7.8)	7.0 (6.0–7.0)	0.642
a′, cm/s	8.0 (7.0–10.0)	9.0 (8.0–10.0)	0.301
E/e′	9.80 (8.44–12.41)	9.20 (7.50–11.29)	0.245
LV-Ed, 1/ml	0.133 (0.100–0.183)	0.127 (0.098–0.162)	0.049
TRV, m/s	2.09 ± 0.38	2.13 ± 0.26	0.469
LAmax volume, ml	88.2 (71.1–119.4)	89.0 (70.2–109.4)	0.225
LAmin volume, ml	43.5 (32.6–56.1)	41.9 (32.6–52.5)	0.122
LA total EF, %	52.1 ± 9.7	52.5 ± 8.2	0.711
LA reservoir fraction, %	106.3 (83.6–146.4)	109.2 (88.4–141.1)	0.811
LA conduit fraction, %	36.8 (24.6–46.8)	27.3 (21.0–33.5)	0.393
LA active EF, %	35.6 ± 9.7	35.7 ± 6.7	0.923
LA-GLS, %	21.0 (17.2–26.9)	19.4 (16.5–24.6)	0.167
LA stiffness index, 1/%	0.46 (0.35–0.58)	0.48 (0.33–0.59)	0.914
%LGE	20.9 (8.5–26.8)	11.1 (7.0–20.0)	<0.001
LGE amount, g	18.2 (4.5–30.6)	10.3 (5.6–17.7)	<0.001
%Transmural LGE, %	20.8 (4.3–27.9)	10.2 (3.6–17.1)	<0.001
Transmural LGE, g	18.2 (4.5–30.6)	9.0 (3.1–15.9)	<0.001
%MVO, %	1.7 ± 4.1	0	<0.001
MVO, g	2.1 ± 5.3	0	<0.001
ECV at initial LGE, %	42.1 ± 6.4	36.4 ± 6.3	<0.001
ECV at initial non-LGE, %	32.8 ± 3.8	28.6 ± 2.2	<0.001

MVO, microvascular obstruction; LV, left ventricular; LVEDV, LV end-diastolic volume; LVESV, LV end-systolic volume; GLS, global longitudinal strain; E, early diastolic inflow velocity; A, late diastolic inflow velocity; DT, deceleration time; s′, systolic septal mitral annular velocity; e′, early diastolic septal annular velocity; a′, late diastolic septal annular velocity; Ed, elastance index; TRV, tricuspid regurgitant velocity; LA, left atrial; EF, emptying fraction; LGE, late gadolinium enhancement; ECV, extracellular volume fraction.

**Table 2:** Comparisons of baseline characteristics and serial structural and functional changes according to microvascular obstruction.

	MVO (+)			MVO (−)		
	(*n* = 31)			(*n* = 41)		
	Baseline	FU	*p*	Baseline	FU	*p*
Age, years	52.1 ± 10.0			57.1 ± 12.5		
Male, *n* (%)	29 (93.5)			39 (95.1)		
LAD/LCx/RCA territory, *n*	22/1/8			27/2/12		
Peak CK-MB level, ug/L	200.6 (69.5–364.2)**			84.9 (28.7–163.5)		
Peak troponin T level, ug//L	5.45 (3.07–14.30)**			2.24 (0.69–4.74)		
Hypertension, *n* (%)	14 (45.2)			17 (41.5)		
Diabetes, *n* (%)	6 (19.4)			8 (19.5)		
Smoking status (non-/ex-/current), *n*	10/12/9			11/17/13		
ARB or ACEi use, *n* (%)	24 (77)			35 (85)		
BB use, *n* (%)	29 (94)			32 (78)		
LV mass, g	126.2 (108.0–144.7)	107.3 (86.6–123.4)	0.001	122.0 (105.2–147.8)	102.4 (81.7–114.4)	<0.001
LV-GLS, %	−12.5 ± 4.5	−13.6 ± 4.1	0.041	−14.3 ± 3.8	−16.0 ± 4.1***	0.002
Regional LS at initial LGE, %	−16.1 ± 5.0	−17.6 ± 4.5	0.026	−17.0 ± 4.8	−18.8 ± 5.5	0.013
Regional LS at initial non-LGE,%	−19.8 ± 6.7	−19.7 ± 6.2	0.941	−22.0 ± 5.4	−22.6 ± 5.7	0.418
Ed, 1/ml	0.17 ± 0.08*	0.14 ± 0.06	0.001	0.14 ± 0.07	0.14 ± 0.08	0.786
e′, cm/s	6.0 (5.0–7.0)	7.0 (6.0–8.0)	0.795	7.0 (5.0–8.0)	6.7 ± 2.4	0.686
E/e′	10.9 (8.8–13.0)	9.1 (8.3–11.5)	0.151	9.1 (8.0–11.1)	9.6 (7.4–11.3)	0.856
RVSP, mmHg	22.1 (16.9–27.3)	22.5 (18.9–25.4)	0.627	24.0 (18.3–28.0)	25.3 (20.3–28.0)	0.774
E/A ratio	1.07 ± 0.34	1.14 ± 0.47	0.522	0.98 ± 0.40	0.97 ± 0.36	0.823
DT, ms	167.4 ± 45.2*	207.0 ± 46.3	0.004	205.3 ± 42.5	241.5 ± 55.9***	<0.001
LA total EF, %	47.9 (44.2–56.5)*	51.3 (44.5–58.4)	0.458	53.4 (48.6–60.5)	53.6 (48.8–59.4)	0.786
LA-GLS, %	19.6 (16.5–24.0)	18.9 (15.0–24.7)	0.728	22.2 (17.7–28.4)	20.4 (17.7–23.6)	0.115
%LGE	26.4 (20.9–34.0)**	15.5 (10.5–23.5)	<0.001	12.9 (4.6–22.3)	8.4 (3.4–14.6)***	<0.001
LGE amount, *g*	26.5 (20.7–44.1)**	16.4 (10.1–23.0)	<0.001	11.0 (3.8–20.8)	7.8 (2.9–13.6)***	<0.001
ECV at initial LGE, %	44.2 ± 4.2**	39.2 ± 5.0	0.002	39.8 ± 7.5	33.5 ± 6.3***	<0.001
ECV at initial non-LGE, %	33.1 ± 4.5	28.8 ± 2.3	<0.001	32.5 ± 3.2	28.5 ± 2.1	<0.001

ARB, angiotensin II receptor blocker; ACEi, angiotensin-converting enzyme inhibitor; RVSP, right ventricular systolic pressure, see abbreviations in Table 1.

* *p* < 0.05.

** *p* < 0.01 compared to the MVO (+) at baseline.

*** *p* < 0.05 compared to the MVO (+) group at follow-up. Some Doppler and T1 mapping parameters were missing during the 9-month follow-up; therefore, the analyses were done in matched cases only.

### Changes in infarct size and LV systolic and diastolic function during follow-up

**Figure 3:**
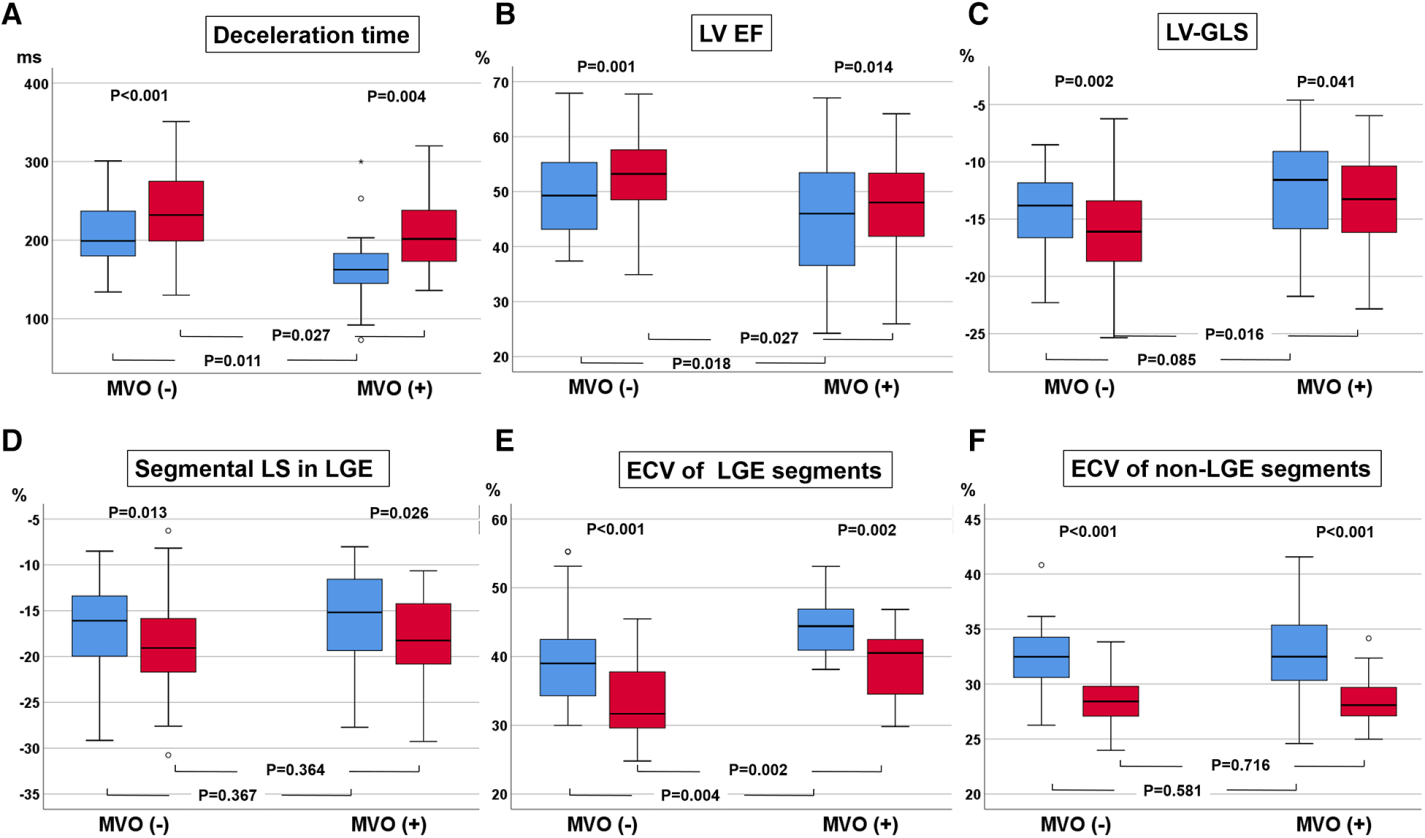
Changes in deceleration time (DT) of early mitral inflow (**A**), left ventricular (LV) ejection fraction (EF, **B**), LV global longitudinal strain (GLS, **C**), regional longitudinal strain (LS, **D**), and extracellular volume fraction (ECV, **E**) in initial late gadolinium enhancement (LGE) segments and ECV at initially non-LGE segments (**F**).

The LVEDVs (*r* = 0.791, *p* < 0.001), LVESVs (*r* = 0.858, *p* < 0.001), and LVEFs (*r* = 0.744, *p* < 0.001) measured in short-axis and long-axis views were significantly correlated with each other. For the analysis, the values from the long-axis view were used. LVEF and LV-GLS were significantly improved over 9 months, from 47.4 ± 9.6% to 51.5 ± 9.5% (*p* < 0.001) and from −13.6 ± 4.2% to −15.0 ± 4.3% (*p* < 0.001), respectively, along with significant decreases in LV mass (124.2–105.9, *p* < 0.001), LVEDV (159 ml–153 ml, *p* = 0.023), and LV-Ed (0.133 1/ml–0.127 1/ml, *p* = 0.049) and an increase in DT of early mitral inflow (188.6 ms–226.3 ms, *p* < 0.001). Regarding infarct size, %LGE amount and absolute LGE mass (g) significantly decreased during the 9-month follow-up, from 20.9 (8.5–26.8)% to 11.1 (7.0–20.0)% and from 18.2 (4.5–30.6) g to 10.3 (5.6–17.7) g, respectively (both *p* < 0.001) (Table 1 and Supplementary Figure S1). All MVO lesions disappeared and were changed to LGE on follow-up CMR (Figure 2). Additionally, both the average ECV of the initial LGE-involved segments (42.1 ± 6.4%–36.4 ± 6.3%, *p* < 0.001) and the average ECV of non-LGE-involved segments (32.5 ± 3.8–28.6 ± 2.2%, *p* < 0.001) significantly decreased during follow-up. Absolute LGE mass at 9 months was inversely correlated with LVEF (*r* = −0.618, *p* < 0.001) and correlated with LV-GLS (*r* = 0.596, *p* < 0.001). Regarding diastolic function, DT (*r* = −0.277, *p* = 0.027) and LA-GLS (*r* = −0.362, *p* = 0.002) were significantly correlated with LGE mass. But, e′, E/e′, LA volume index, and TRV were not significantly correlated with LGE mass. The average longitudinal strain (LS) of LGE-involved segments was significantly improved (from −16.6 ± 4.9% to −18.3 ± 5.1%, *p* = 0.001), while there was no significant change in non-LGE-involved segments. The LVEF, LV-GLS, and LS of LGE segments were significantly improved in both MVO presence and absence groups, but the degree of improvement was less prominent in the MVO group. The degree of DT prolongation during follow-up was less prominent in the MVO group (Figure 3). However, in both groups, both ECVs of initial LGE segments and non-LGE segments decreased during the 9-month follow-up (Table 2).

### Relationship between changes in infarct size and LV function

Regarding diastolic functional indexes, changes in absolute LGE amounts (g) or percent LGE were weakly but significantly correlated with LV-Ed (*r* = −0.317, *p* = 0.011) or LA total emptying fraction (*r* = 0.269, *p* = 0.027), but not with conventional parameters, such as e′, E/e′, LA volume index, and TRV. In addition, neither changes in LVEF, regional LGE-involved segmental LS, nor LV-GLS were correlated with LGE mass and ECV changes. Regarding segments with >50% transmural LGE, initial transmural LGE segment numbers significantly decreased from 2.2 ± 2.1–1.1 ± 1.4, *p* < 0.001. However, the degree of improvement therein was not significantly correlated with improvement in LVEF and LV-GLS. Also, neither ECVs of non-LGE segments nor LGE-involved segments were related to the degree of improvement in LVEF and LV-GLS (Supplementary Figure S2).

### Relationship between LV systolic and diastolic functional improvement

**Figure 4:**
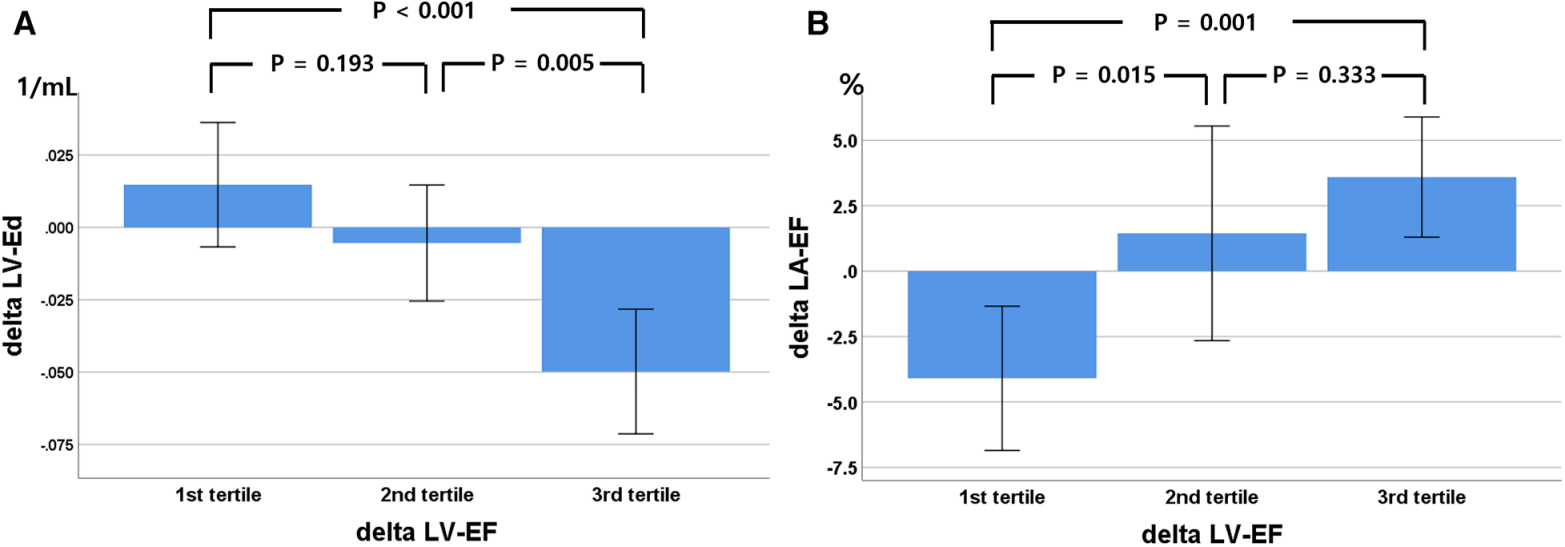
Relationship between changes in left ventricular (LV) ejection fraction (EF), divided into three tertiles, with LV elastance index (Ed, **A**) and left atrial (LA) total emptying fraction (**B**) during the 9-month follow-up. Delta, change in value over 9 months.

Although most patients exhibited improvements in LV systolic function (LVEF and LV-GLS), the distribution of the diastolic functional grade was not changed, likely because almost all patients had grade I diastolic dysfunction according to the current guidelines (14, 15). Among the three patients with grade II diastolic dysfunction, two patients changed to grade I diastolic dysfunction due to a decrease in E/e′ or TRV. Among the diastolic functional indexes, only DT and LV-Ed, reflecting LV stiffness, improved during the follow-up period. The degree of LVEF and change in LV-GLS were not significantly related to improvements in conventional echocardiographic LV diastolic functional indexes. However, changes in LA total emptying fraction (*r* = 0.279, *p* = 0.019) and LV-Ed (*r* = −0.494, *p* < 0.001) were significantly correlated with the degree of change in LVEF (Figure 4).

## Discussion

After successful revascularization, we found that LV systolic function significantly improved, along with a decrease in LGE and LV mass over 9 months. In contrast, conventional LV diastolic functional indexes did not significantly change during the follow-up period. However, shortened DT, LA phasic function, and LV-Ed significantly improved in accordance with LV systolic functional improvement, suggesting sensitive LV diastolic functional indexes in the acute phase of reperfused AMI. Their improvements in systolic and diastolic function occurred even in patients with MVO; however, the degree of improvement was less prominent in patients with MVO.

### Serial structural and functional changes after successful revascularization in AMI

We found that after successful revascularization, infarct size and the presence of MVO were significantly correlated with systolic functional indexes. Shortened DT of early mitral inflow, increased LV-Ed, and impaired LA phasic function, suggesting increased LV stiffness, were significantly correlated with the presence of MVO and infarct size within 7 days after revascularization. During the 9-month follow-up, shorted DT and higher LV-Ed, reflecting acute increases in LV stiffness, were significantly improved, but were not correlated with other diastolic functional indexes. During the follow-up period, absolute LGE amount, % LGE, and LV mass significantly decreased in accordance with improvements in LVEF and LV-GLS. Our results were consistent with those of the previous studies (2). According to these study results, the current LGE quantification technique in the acute stages of myocardial infarction tends to include a mixture of viable and non-viable myocardium, which means that the LGE area measured by the full-width at half-maximum method does not always represent the absolute scar (3). These findings are also supported by a decrease in ECV during the follow-up period in both initial LGE-involved and non-LGE-involved segments. An interesting finding was that ECV decreased during follow-up even in remote myocardium. However, inconsistent with previous studies (16), we could not find any effects of remote myocardial ECV changes on LV remodeling and systolic function. The degree of LGE amount change was not significantly correlated with the LV systolic and diastolic functional changes, suggesting multifactorial influences.

### Effects of MVO on serial structural and functional changes

All the MVOs have been known to become scar after 3 months and related to adverse remodeling in several previous studies (17). Our study results were also consistent with those of previous studies, as all of the MVO lesions changed to the LGE area. Patients with MVO experienced less improvement in LVEF and LV-GLS accompanied by less LV mass reduction. They also experienced less improvement in regional myocardial function and ECV in the initial LGE area during the follow-up. A possible explanation is that the initial LGE area can include a mixture of scar and edema (or a mixture of viable and non-viable tissue). Therefore, in some parts, edema tends to disappear over time, and LV mass can be decreased after the initial AMI period. However, since patients with MVO tend to have a higher non-viable portion, the effects of LV mass reduction and LV systolic functional improvement during the 9-month follow-up were less prominent. An interesting finding is that DT was more prominently increased during follow-up in the MVO group, suggesting that in the acute phase of myocardial infarction, MVO transiently increases LV stiffness (shortens DT) and then prolongs LV relaxation over time. Our study results support the previous study, which showed that DT was a sensitive and reliable index of transient increase in LV filling pressure and stiffness in AMI (18, 19). Therefore, patients with MVO in AMI would be vulnerable to developing pulmonary edema in response to preload augmentation. These patients may need to use nitrate or low-dose diuretics to prevent pulmonary edema. Regarding microstructural changes, the LGE area with MVO experienced less improvement in ECV. Therefore, it is a potential source of progressive remodeling and arrhythmic substrate. One reason for heterogeneous structural and functional change, even in the MVO group, might be concomitant intramyocardial hemorrhage; however, we could not confirm its presence with T2* images in this study (20). The effects of MVO on remote myocardium were not significant.

### Discrepancy in the improvement of LV systolic and diastolic functional indexes

Changes in LV-Ed and LA total emptying fraction by CMR were significantly correlated with changes in LVEF, suggesting a more sensitive index for diastolic functional changes than the current conventional four echo-Doppler components. In addition, LA total emptying fraction improvement was in accordance with the decrease for LGE over 9 months. A possible explanation of phasic LA total emptying fraction and LV-Ed as sensitive LV diastolic functional indexes is that they reflect the final product of delayed LV relaxation or increased LV stiffness and contribute to stroke volume. Recent studies have shown that LA phasic function or GLS can provide incremental information on LV diastolic function in various diseases, especially in patients with HF with preserved EF (21). We can also infer that LV chamber diastolic function is affected not only by infarcted myocardium but also by compensatory remote myocardium, which is suggested by the fact that almost all AMI patients have relaxation abnormality of LV filling and some patients even have normal filling patterns, especially young patients, despite a large infarct size (6). We previously showed that age, underlying long-standing cardiac risk factors, and remote myocardial function contribute to LV chamber diastolic function in AMI patients (6). Therefore, conventional echo-Doppler parameters are not sufficient to follow sensitive changes in global LV function; rather, LV global GLS may provide more information for combined LV systolic and diastolic function. A sophisticated and more sensitive regional and global diastolic functional index is needed to stratify vulnerable patients.

### Study limitations

Although all patients underwent repeated CMR and echocardiography at around 9 months after baseline evaluation, there were some differences in the timing of CMR and echocardiography. For the baseline study, the mean interval was 2.2 ± 6.1 days, and for the follow-up study, the mean interval was 2.5 ± 2.3 months. As a diastolic functional index, LV diastolic global or segmental strain rates were not measured due to limitations in temporal resolution in cine imaging. Due to the small number of study subjects in this study, some important relationships could not reach statistical significance. Various indexes, from direct measurements or multiple calculations, were used in this study. In addition, there were some missing values in ECV. Therefore, there was a possibility that small measurement errors might be exaggerated in the final analysis. This might interpret analysis results more complex.

## Conclusion

Although conventional echo-Doppler-derived diastolic functional indexes were not related to infarct size and LV systolic functional changes, indexes of LV stiffness (such as DT and Ed) or LA phasic functional indexes were correlated with them, suggesting more sensitive indexes in this group of patients. This suggests that mitral inflow-derived stiffness and pressure volume-related LV indexes or LA phasic function are more sensitive indexes reflecting LV diastolic functional status than conventional ASE/EAE recommended indexes in AMI patients. In addition, the degrees of improvement therein were less prominent in patients with MVO.

## Data Availability

The raw data supporting the conclusions of this article will be made available by the authors, without undue reservation.
