# Supplementary material for: Determinants and effects of microvascular obstruction on serial change in left ventricular diastolic function after reperfused acute myocardial infarction
Source: Front Cardiovasc Med. 2024 Apr 26;11:1338940. doi: 10.3389/fcvm.2024.1338940 (PMC11100411; doi:10.3389/fcvm.2024.1338940)
Supplement: Supplementary file 1 [file Table1.docx]

**Supplemental table 1.** Formulas for the calculations of each study parameter

| **Parameters** | **Formulas** |
| --- | --- |
| **LA total emptying fraction** | (LA maximal volume - LA minimal volume) / LA maximal volume |
| **LA reservoir fraction** | (LA maximal volume - LA minimal volume/LA minimal volume) |
| **LA conduit fraction** | (LA maximal volume - LA pre-A volume) / LA maximal volume |
| **LA active emptying fraction** | (LA pre-A volume - LA minimal volume) / LA pre-A volume |
| **LA stiffness index** | LA-GLS/(E/e’) |
| **LV end-diastolic elastance (LV-Ed)** | E/e’/ LV stroke volume by CMR |

LA, left atrial; GLS, global longitudinal strain; LV, left ventricular; Ed, elastance

**Supplemental table 2.** Baseline clinical and laboratory findings

| **Variables** |  |
| --- | --- |
| **Age**, years | 55.0±11.7 |
| **Male**, n(%) | 68 (94) |
| **LAD/LCx/RCA territory**, n | 49/3/20 |
| **Peak CK-MB level**, ug/L | 107.5 (43.8-240.8) |
| **Peak troponin T level**, ug/L | 3.79 (0.86-7.82) |
| **Hypertension**, n(%) | 31 (43) |
| **Diabetes**, n(%) | 14 (19) |
| **Smoking status (Non-/Ex-/ Current)**, n | 21/29/22 |
| **Body surface area**, m^2^ | 1.82±0.16 |
| **Systolic blood pressure at CMR**, mmHg | 113.5 (104.0-132.0) |
| **Diastolic blood pressure at CMR**, mmHg | 73.2±11.2 |
| **Atrial fibrillation**, n(%) | 2 (3) |
| **eGFR**, ml/m^2^ | 90.8 (78.4-107.5) |
| **ACEI/ARB**, n(%) | 59 (82) |
| **BB**, n(%) | 61 (85) |
| **Diuretics**, n(%) | 5 (7) |

LAD, left anterior descending artery; LCx, left circumflex artery; RCA, right coronary artery; CK, creatinine kinase; CMR, cardiovascular magnetic resonance imaging; eGFR, estimated glomerular filtration rate; ACEI, angiotensin-converting enzyme inhibitor; ARB, angiotensin receptor blocker; BB, beta-blocker
