# Supplementary figures and images for: Determinants and effects of microvascular obstruction on serial change in left ventricular diastolic function after reperfused acute myocardial infarction
Source: Front Cardiovasc Med. 2024 Apr 26;11:1338940. doi: 10.3389/fcvm.2024.1338940 (PMC11100411; doi:10.3389/fcvm.2024.1338940)

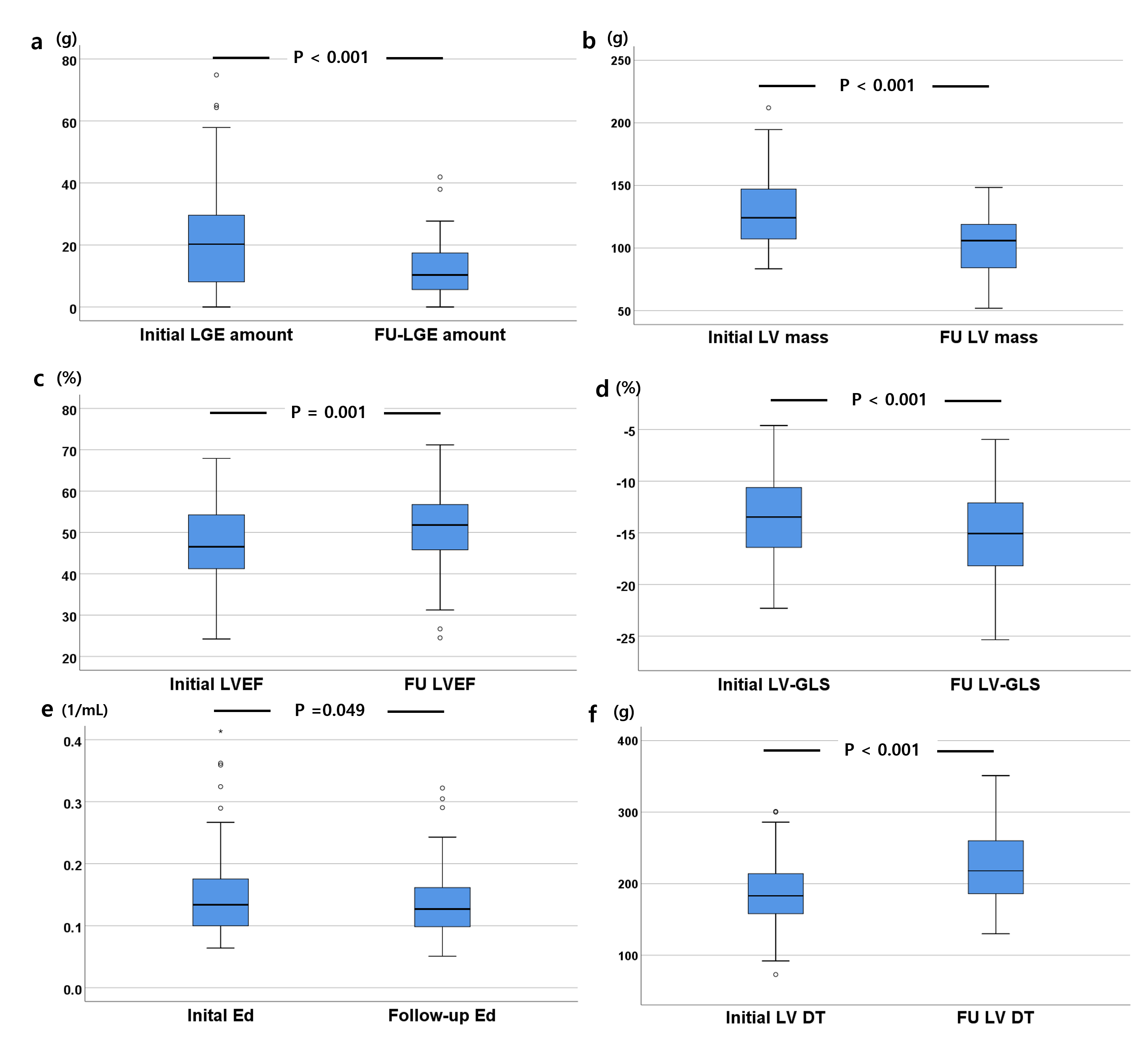

Supplement: Supplementary file 2 [file Image1.tif]

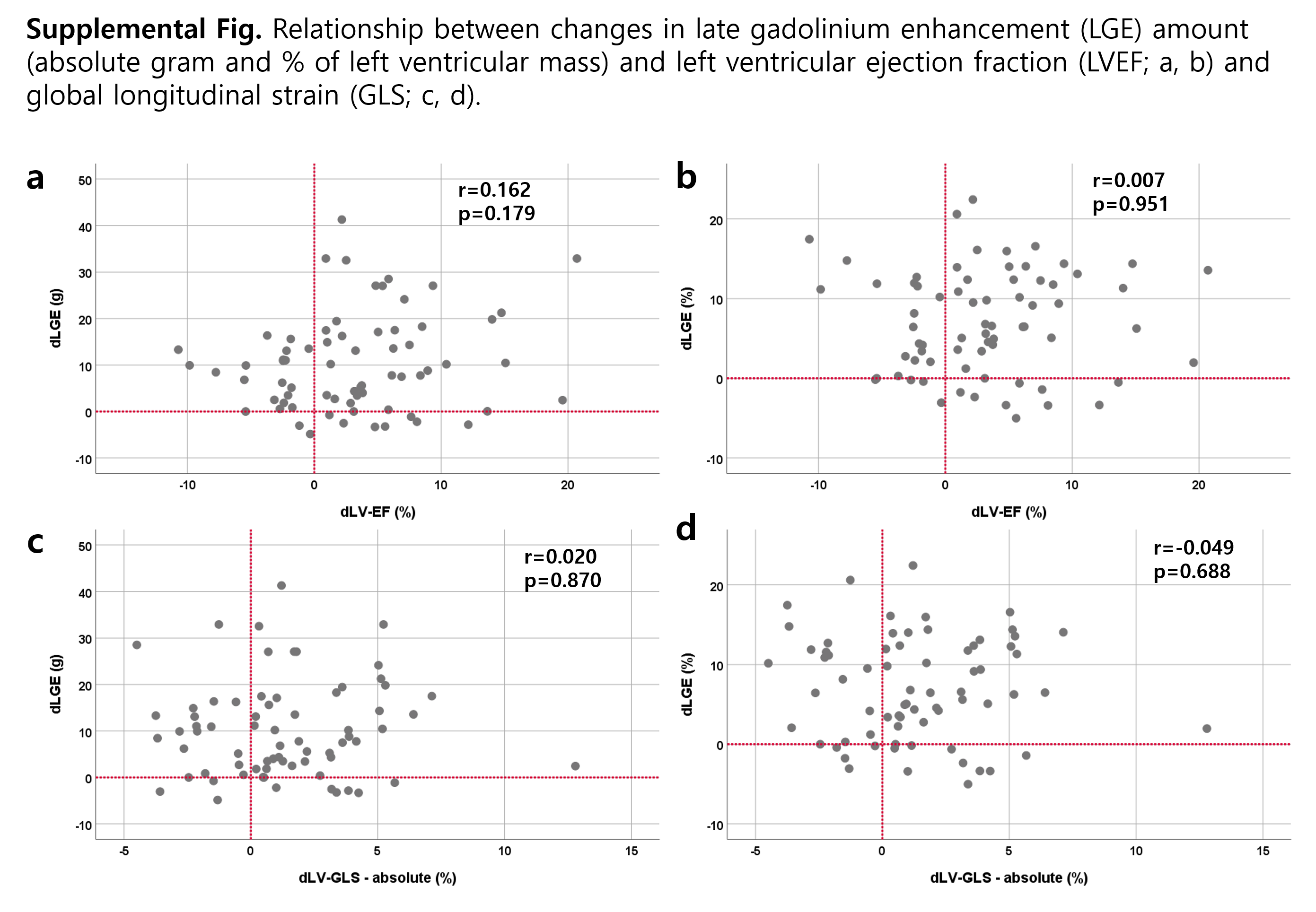

Supplement: Supplementary file 3 [file Image2.tif]
